# Influence of the reactor environment on the selective area thermal etching of GaN nanohole arrays

**DOI:** 10.1038/s41598-020-62539-1

**Published:** 2020-03-27

**Authors:** Pierre-Marie Coulon, Peng Feng, Benjamin Damilano, Stéphane Vézian, Tao Wang, Philip A. Shields

**Affiliations:** 1 Department Electrical & Electronic Engineering, University of Bath, Bath, BA2 7AY UK; 2 Department of Electronic and Electrical Engineering, University of Sheffield, Sheffield, S1 4DE UK; 3 Université Côte d’Azur, CNRS, CRHEA, rue B. Gregory, 06560 Valbonne, France

**Keywords:** Materials science, Synthesis and processing, Optical materials and structures

## Abstract

Selective area thermal etching (SATE) of gallium nitride is a simple subtractive process for creating novel device architectures and improving the structural and optical quality of III-nitride-based devices. In contrast to plasma etching, it allows, for example, the creation of enclosed features with extremely high aspect ratios without introducing ion-related etch damage. We report how SATE can create uniform and organized GaN nanohole arrays from *c*-plane and (11–22) semi-polar GaN in a conventional MOVPE reactor. The morphology, etching anisotropy and etch depth of the nanoholes were investigated by scanning electron microscopy for a broad range of etching parameters, including the temperature, the pressure, the NH_3_ flow rate and the carrier gas mixture. The supply of NH_3_ during SATE plays a crucial role in obtaining a highly anisotropic thermal etching process with the formation of hexagonal non-polar-faceted nanoholes. Changing other parameters affects the formation, or not, of non-polar sidewalls, the uniformity of the nanohole diameter, and the etch rate, which reaches 6 µm per hour. Finally, the paper discusses the SATE mechanism within a MOVPE environment, which can be applied to other mask configurations, such as dots, rings or lines, along with other crystallographic orientations.

## Introduction

Nanostructures are continuously drawing the attention of the III-nitride community for their success in improving light emitting devices^1–4^ and their use for emerging applications such as water splitting^5^, single photon sources^6^, piezoelectric nanogenerators^7^ or solar light harvesting^8^. Among the different geometries available, arrays of nanoholes, in the form of a porous or two-dimensional photonic crystal (2D PhC) layer, have been widely investigated. On one side, a porous layer can provide a change in refractive index^9,10^, an increased surface area resulting in both higher sensitivity^11^ and larger photocurrent^12,13^, a more efficient light extraction due to enhanced scattering^14,15^, and improved crystal quality by reducing strain and dislocation density^16–19^. On the other side, a 2D PhC layer allows one to enhance the IQE thanks to the Purcell effect, increase the light extraction efficiency and improve/control the directionality of III-nitride based light emitting diodes (LED)^20–23^. As such, networks of nanoholes have been used in several applications such as distributed Bragg reflectors^10^, sensors^11^, hydrogen generation^12,13^, LEDs^14,22,23^, free-standing GaN films^17^, and energy storage^18,19^.

The fabrication of nanohole arrays is usually achieved from a III-nitride layer or an LED structure via a subtractive process or what is called a top-down approach. Porous layers are generally obtained from GaN layers by various techniques, such as electrochemical etching^9,11,14,16^, photo-electrochemical etching^12,13^, metal assisted chemical etching^24,25^, and high temperature annealing with^26^, or without, a catalyst^15,17–19,27,28^. Although the depth of the pores can reach a few microns^13,17–19,24,25^, the resulting layers are generally composed of a self-organized network of pores, with a poor control on the dimensions, the shape and the density. In addition, chemicals involved in some of the porosification processes can have an impact on the performance of the device, which can explain why the porosification mainly focuses on GaN layers rather than a full LED structure. In order to precisely control the diameter and density of the nanoholes, a nanotextured mask with a well-defined and uniform configuration needs to be applied. Yet, in the case of the aforementioned porosification approaches that rely on chemical etching or thermal etching, the presence of a mask doesn’t guarantee that the initial pattern will be selectively transferred to the underlying GaN layer^27^. Indeed, the porosification mechanism of these approaches often depends on the intrinsic characteristics of the GaN layer, such as the doping, the threading dislocation or the pit density^10,12–14,16,17,19,24,27^. Dry etching of GaN, on the contrary, is not impacted by those characteristics and, in addition, shows a fairly good anisotropy when covered by a hard etch mask such as SiO_2_, SiN_x_ or Ni. Thus, several techniques such as inductively coupled plasma (ICP) etching have been employed, together with etch masks, to create nanoporous GaN films^29^, but mainly to achieve 2D PhC on the surface of a LED structure^20–23^. In that case, the configuration of the nanoholes can be accurately tuned and controlled by various nanopatterning techniques, such as electron beam lithography, interference lithography or nanoimprint lithography. However, controlling the nanohole profile and achieving high etching anisotropy with straight sidewall remains challenging^21,30^. Compared to dry etching of large micrometre size structures, or even of nanorods, dry etching of high aspect ratio nanoholes is more sensitive to the  shadowing effect of neutral species, electrostatic deflection of incoming ions in the holes, mask erosion or the difficulty in removing etch by-products^21,30^. Although the parameters of the ICP chamber can be optimized to reach fairly straight nanohole sidewalls for an etch depth up to hundreds of nanometers^21,30^, deeper etching over a micron will be inevitably limited by the selectivity, the thickness of the mask and aspect ratio dependent etching, which induces a dramatic reduction in etch rate for deep holes. In consequence, the profile of high aspect ratio nanoholes will be degraded. Hence this remains an issue to be solved^30^.

As a solution to circumvent dry etching limitations, thermal etching of GaN appears to be the most promising route to achieve highly organized nanoholes with a high aspect ratio and straight sidewall profile. Compared to dry etching, thermal etching doesn’t generate any ion-induced surface defects that can affect the optical properties. The etch depth is also not limited by the thickness of the mask, providing that the layer employed is thermally stable to the annealing temperature, hence resulting in an infinite selectivity to GaN. Finally, under appropriate annealing conditions, the etching rate can be highly anisotropic, with a preferential etching of the *c*-plane compared to the sidewall. Indeed, Miao *et al*. reported the formation of a nanoporous GaN template in a metal organic vapor phase epitaxy (MOVPE) chamber from a SiO_2_ nanohole mask^27^. In this work, nanoholes with a height over a micron and with a fairly straight sidewall profile were obtained. However, the process was reported to be strongly sensitive to threading defects, resulting in the formation of pores under the mask and, therefore, with a poor organization of nanoholes overall and poor uniformity of the nanohole dimensions. More recently, Damilano and co-authors reported several works on the selective area sublimation (SAS) of nanorods and nanoholes having a straight sidewall profile, achieved in a molecular beam epitaxy (MBE) chamber either from a SiN_x_ self-organized mask^15,31^, or a SiN_x_ nanopatterned mask^32,33^. With their conditions, nanorods and nanoholes with a high organization and fairly uniform dimensions were successfully achieved^32,33^. Despite the successful formation of nanoholes, few technical details have been provided on the impact of the thermal etching environment on the morphology of the nanohole arrays. Compared to MBE experiments performed under high vacuum, a MOVPE reactor provides a broader range of parameters to be investigated, with the potential of achieving a higher thermal etch rate. Moreover, MOVPE reactors are currently at the core of most III-nitride LED manufacturing processes enabling commercial scale-up. As such, a thermal etching process on GaN layers could be easily implemented in a commercial MOVPE growth reactor as a simple annealing step prior to the regrowth of 2D or 3D layers.

In this work, the impact of the thermal etching environment on the SATE of GaN is investigated within a MOVPE reactor. Parameters such as the temperature, the pressure, the supply of NH_3_ and the carrier gas mixture are explored. The morphology of the nanoholes and their characteristics, such as the uniformity of diameter, the shape and the height were characterized after etching using a scanning electron microscope (SEM). It is found that, among other parameters, the supply of NH_3_ during thermal etching plays a crucial role in obtaining hexagonally faceted nanoholes. Optimized annealing conditions are successfully employed on *c*-plane and $(11\bar{2}2)$ semi-polar GaN templates having various mask configurations to achieve high aspect ratio faceted nanoholes. Finally, the etching mechanism behind the SATE of GaN nanohole arrays within a MOVPE environment is discussed.

## Results and Discussion

To create GaN nanoholes, a SiN_x_ mask having circular nano-openings was fabricated by Displacement Talbot Lithography (DTL)^34^ and inductively coupled plasma etching on GaN templates. A commercial ~7 µm *c*-plane GaN template and a ~1.5 µm $(11\bar{2}2)$ semi-polar GaN template, both grown by MOVPE respectively on (0001) and r-plane sapphire substrates, were used for the experiments. After the DTL process, a hexagonal array of circular openings was created in ~30 nm thick SiN_x_. A parametric thermal etching investigation was performed on samples with mask openings that have a relatively large diameter of ~590 nm and a pitch of 1.5 µm. Optimized thermal etching parameters were then used for further experiments on various mask configurations created on *c*-plane and $(11\bar{2}2)$ semi-polar GaN templates. A detailed description of the dielectric mask fabrication process can be found in the Methods section and our previous publication^33^, while SEM images of the SiN_x_ openings are shown in Fig. S1. After careful cleaning of the wafer, the GaN patterned sample was placed in a MOVPE reactor for SATE experiments.

**Figure 1:**
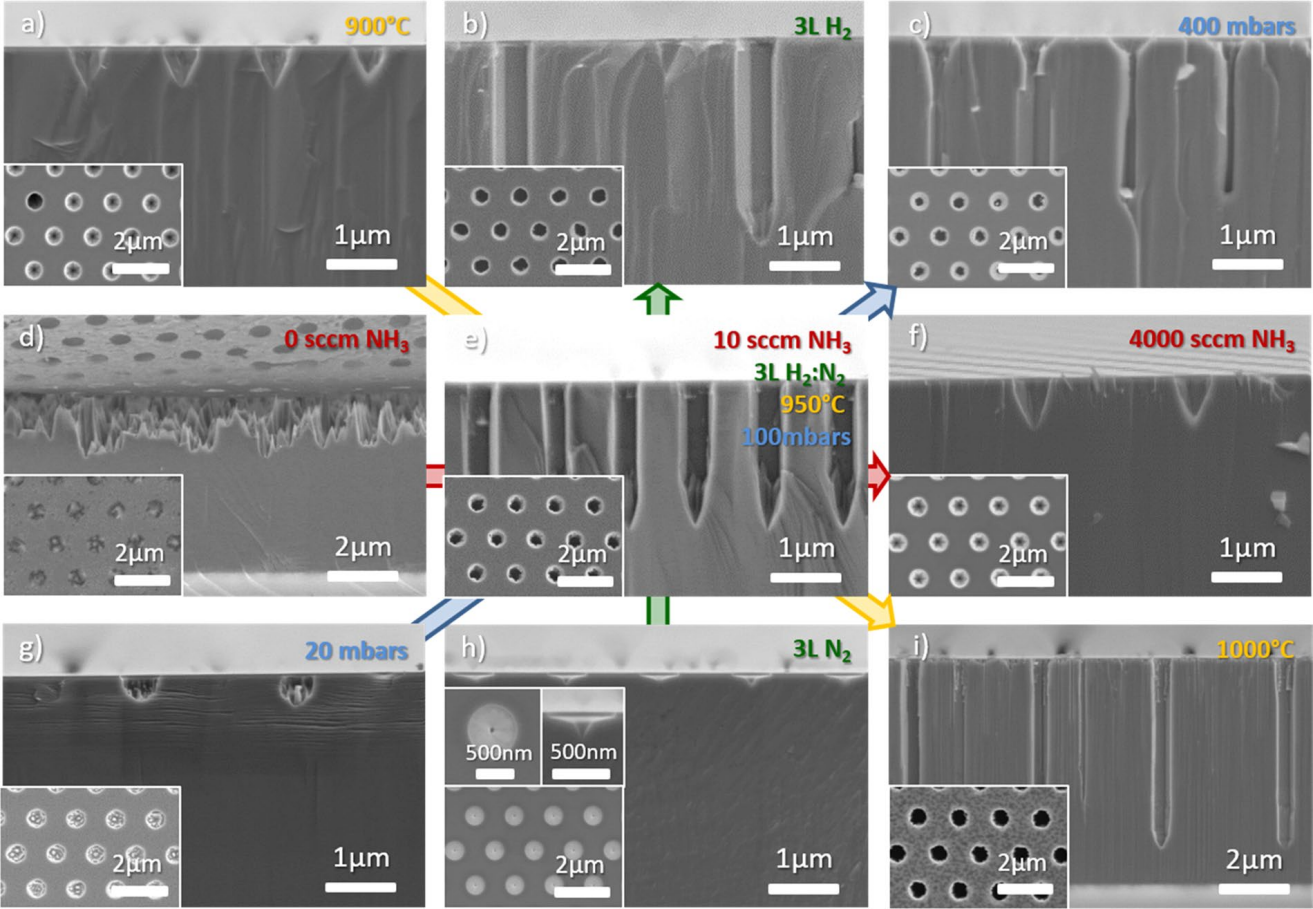
Cross-section and related plan view (in inset) SEM images of GaN nanoholes after 1 h SATE. All images correspond to the four annealing parameters given in (**e**), unless specified on the image.

To study the influence of several annealing parameters, namely the NH_3_ flow rate, the proportion of carrier gas, the temperature and the pressure, four sets of experiments in which one parameter was varied, have been performed on *c*-plane GaN template. In this investigation, the annealing time was fixed to one hour. Figure 1 displays the scanning electron microscopy (SEM) plan-view (in inset) and cross-section images of the resulting GaN nanoholes for the various thermal etching experiments, with the central one in Fig. 1e being in common with the four sets of experiments. Table 1 in the Methods section gives further details about the annealing conditions employed and the related nanohole characteristics such as the *c*-plane etch depth, uniformity of the nanohole diameter or the formation or not of non-polar facets.

**Table 1:** Thermal etching conditions and nanohole characteristics for the experiments presented in Fig. 1.

Fig. nb	Temperature (°C)	Pressure (mbars)	NH_3_ flow rate (sccm)	H_2_:N_2_ carrier gas (L)	c-plane etch depth ± 50 nm (nm)	Uniformity of the GaN opening	Non-polar faceted sidewalls
1.a	900	100	10	3.2: 0.8	550	no	no
1.b	950	100	10	4: 0	3000	no	yes
1.c	950	400	10	3.2: 0.8	2300	no	yes
1.d	950	100	0	3.2: 0.8	3000–3500	yes	no
1.e	950	100	10	3.2: 0.8	2100	no	yes
1.f	950	100	4000	3.2: 0.8	700	no	no
1.g	950	20	10	3.2: 0.8	500	yes	no
1.h	950	100	10	0: 4	150	no	no
1.i	1000	100	10	3.2: 0.8	5900	yes	yes
2	975	50	10	4: 0	2300	yes	yes

### Effect of NH_3_

Figure 1d–f display SATE experiments as a function of the NH_3_ supply. Without NH_3_, the GaN layer is thermally etched along the vertical and lateral directions, resulting in a poor anisotropy and a rough GaN surface on which the SiN_x_ mask collapsed (Fig. 1d). The poor anisotropy is already clearly observed after 10 min (Fig. S2). For 10 sccm NH_3_, the thermal etching conditions are highly anisotropic with the formation of vertical non-polar faceted sidewalls (Fig. 1e). At high NH_3_ flow rate of 4000 sccm, the etch depth drastically decreases and inclined facets are formed rather than non-polar ones (Fig. 1f). Thus, in the annealing conditions employed, the supply of NH_3_ is a critical parameter that helps to control first, the anisotropy of the etching and second, the formation of vertical non-polar or inclined semi polar facets.

### Effect of carrier gas

Figure 1b,e,h show the impact of carrier gas. Under pure N_2_, the *c*-plane GaN surface is found to be fairly stable with almost no thermal etching occurring in the mask openings (Fig. 1h). Indeed, after one hour, a pit can be merely observed in the middle of each opening (insets in Fig. 1h). When N_2_ is substituted by H_2_, the thermal etch rate is highly increased (Fig. 1e), to reach a maximum value under pure H_2_ (Fig. 1b). Therefore, to increase the aspect ratio of nanoholes pure H_2_ should be employed.

### Effect of temperature

**Figure 2:**
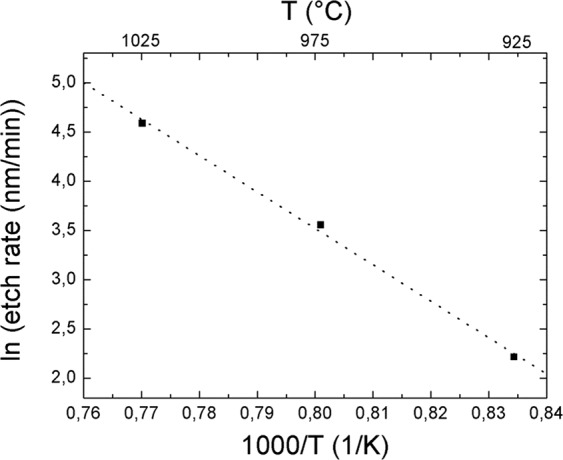
Arrhenius plot of the GaN decomposition rate within SiN nano-openings as a function of temperature in log scale. The fitting by a linear function gives an activation energy of 3.07 eV.

Figure 1a,e,i illustrate the evolution of the nanoholes as a function of the temperature. At low temperature, the nanoholes possess a low etch depth and display an inclined sidewall profile (Fig. 1a), similarly to the SATE experiments performed under high NH_3_ flow rate. As the temperature increases, vertical non-polar facets are formed along with a significant gain in etch depth (Fig. 1e). For the highest temperature investigated, the vertical etch rate almost reaches 6 µm per hour resulting in nanoholes having an aspect ratio of 10 (Fig. 1i). In addition, the overall surface of the *c*-plane GaN openings appears to be etched away (inset in Fig. 1i), which was not the case for lower temperature. Further, at high temperatures, a randomly distributed change in contrast can be observed below the SiN_x_ mask (inset in Fig. 1i) along with the formation of additional nanoholes in between the main 1.5 µm pitch periodic array (Fig. 1i). This observation suggests that high temperature thermal etching becomes more dislocation sensitive^17,27,35^, inducing the formation of nanopits/nanoholes below the SiN_x_ mask. Finally, by extracting the *c*-plane etch rate as a function of temperature in log scale, as shown in Fig. 2, and fitting a linear function (dashed line), an activation energy, E_A_, of 3.07 eV has been extracted. This value agrees quite well with previous reports^36,37^.

### Effect of pressure

Figure 1c,e,g finally show the impact of pressure on SATE experiments. For a relatively high pressure, anisotropic vertical etching occurs with the formation of vertical non-polar sidewalls (Fig. 1c). However, it can be observed that the diameter of the nanoholes within each GaN opening is highly non-uniform (inset in Fig. 1c). Decreasing the pressure helps to improve the uniformity (inset in Fig. 1e) to achieve fairly uniform etched GaN circular openings for the lowest pressure (inset in Fig. 1g). Yet, the drawback of using low pressure is a serious decrease in vertical etch rate and the absence of well-defined non-polar facets.

**Figure 3:**
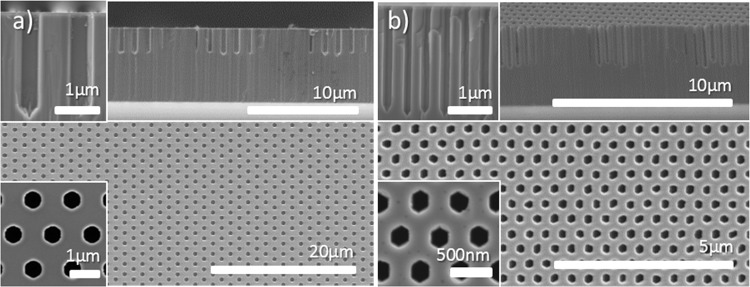
Cross-section and plan-view images of GaN nanohole arrays for (**a**) 1.5 µm pitch after 1 h SATE (~2.35 µm etch depth) and (**b**) 500 nm pitch after 2 h SATE (~2.4 µm etch depth). Inset are high magnification SEM images.

Following the parametric study, SATE conditions have been tuned to achieve uniform openings, non-polar faceted sidewalls and low thermal evaporation below the SiN_x_ mask. Figure 3a,b display the results obtained for two configurations of nano-openings in SiN_x_ having a pitch of 1.5 µm and 500 nm, respectively. The nanoholes are fully open without any GaN remaining at the periphery of the SiN_x_ mask (lower inset in Fig. 3a,b) and are uniform, both in diameter and height. Plan-view and cross-sectional images indicate the formation of well-defined and straight hexagonal facets, with only few GaN desorption residues at the junction between the non-polar planes (upper inset in Fig. 3a). Careful optimisation of the cooling down step or a short GaN regrowth step should limit the formation of such residues, as suggested by regrowth experiments (see Fig. S6). Finally, no thermal etching seems to occur under the SiN_x_ mask. Note that the distortion of the hexagonal shape observed for the 500 nm pitch is more likely to be due to the initial small deformation of the nanohole opening in SiN_x_ (see Fig. S1b). Although not rigorously investigated, the etch rate appears to depend on the feature aspect ratio, likely due to the more difficult supply of H_2_ and NH_3_ and removal of the etch by-products. Note that the flat of the sapphire wafer is found to be parallel to the faceted sidewalls. Given that the flat of the sapphire is parallel to the $(11\bar{2}0)$ *a*-plane, and that the GaN hexagonal lattice is rotated by 30° along the *c*-axis with respect to the sapphire unit cell^38^, the six non-polar facets can be identified as $(1\bar{1}00)$ *m*-planes.

**Figure 4:**
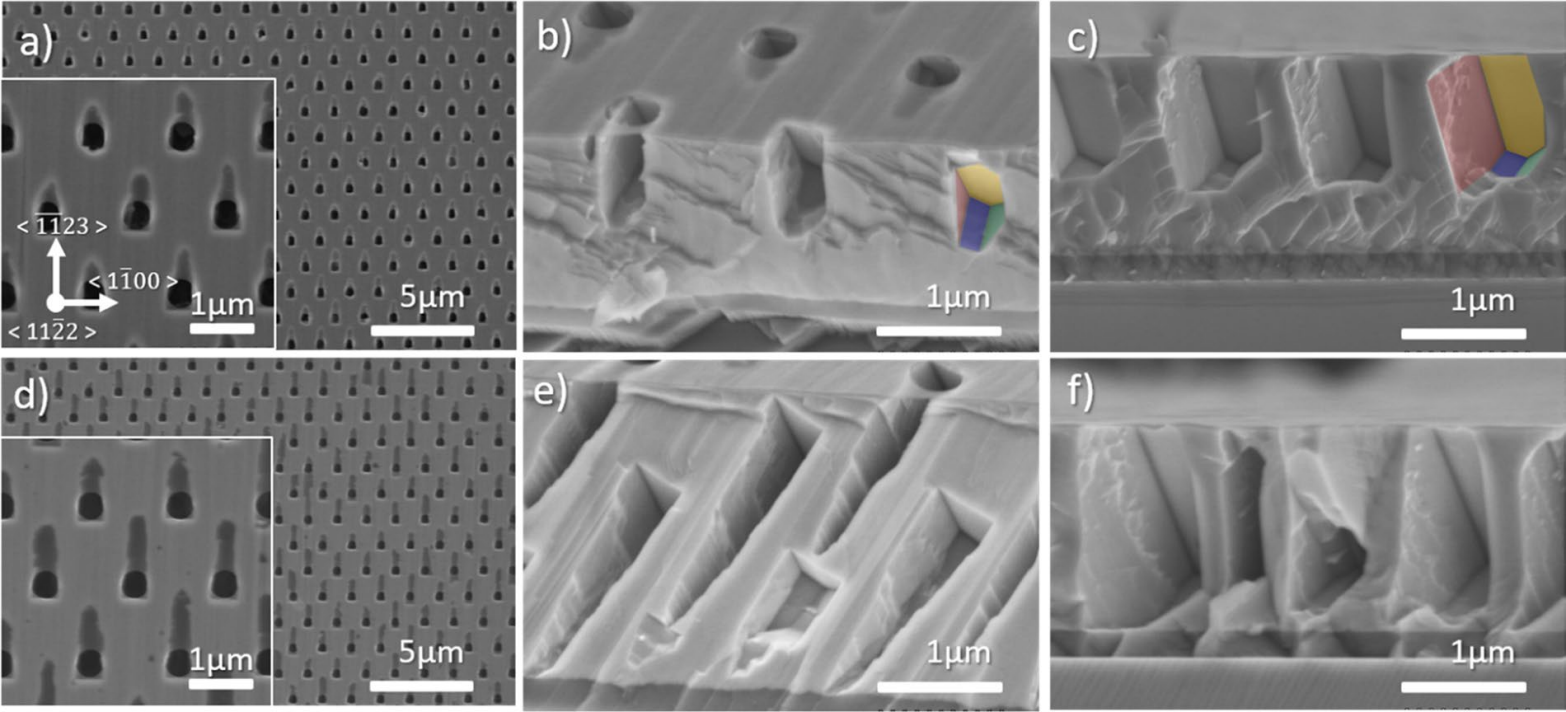
(**a**,**d**) Plan view, (**b**,**e**) 45° tilt and (**c**,**f**) cross-section SEM images of $(11\bar{2}2)$ semi-polar GaN nanoholes after 30 (upper row) and 60 min (lower row) SATE. Inset are high magnification plan view SEM images. The color code employed to identify the facets highlighted in b and c is the same as the one employed in Fig. 5b.

In addition to *c*-plane GaN layers, SATE experiments have also been carried out on $(11\bar{2}2)$ semi-polar GaN layers, which are displayed in Fig. 4a–c,e,f, for a time of 30 and 60 min, respectively. Plan-view images reveal three contrasts: the mostly unetched area in light grey, the circular thermally etched area within the SiN_x_ opening in black, and a trail going from the opening toward the $\langle \overline{11}23\rangle$ direction (inset in Fig. 4a) in dark grey. Tilted-view and cross-section SEM images reveal first, the formation of well-defined facets within the nanoholes, second, that the dark grey trail is related to thermal etching occurring below the SiN mask, and third, that the buffer layer is reached after 60 min.

**Figure 5:**
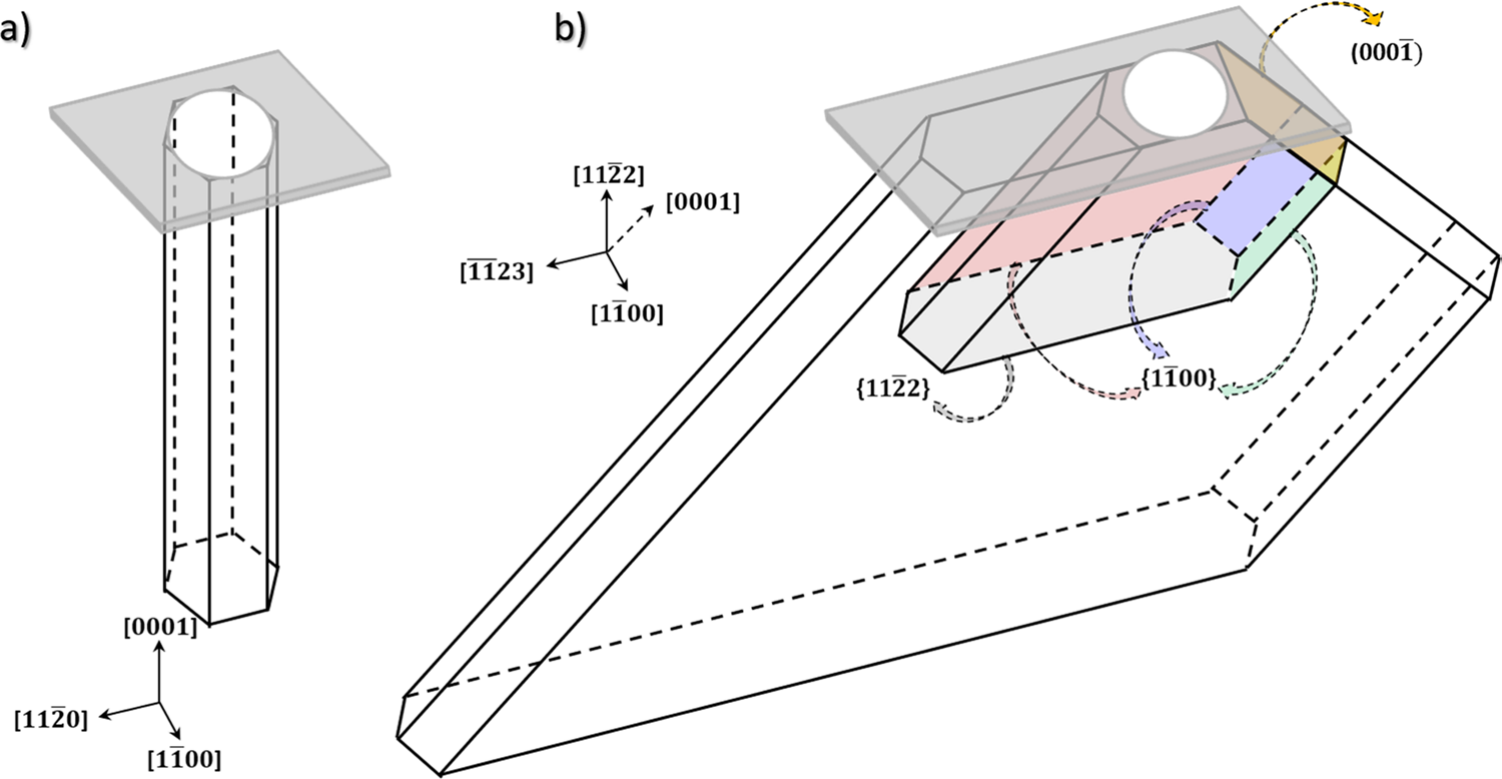
3D schematic of the nanohole geometry for (**a**) *c*-plane and (**b**) $(11\bar{2}2)$ semi-polar GaN layers. The color code employed to identify the facets highlighted in b is the same as the one employed in Fig. 4b,c.

To better visualize and identify the various nanohole facets formed after thermal etching of $(11\bar{2}2)$ semi-polar GaN layers, a three-dimensional schematic of the nanohole geometry has been extrapolated and is displayed in Fig. 5b. Figure 5a represents the 3D hexagonal shape of the nanohole obtained from *c*-plane GaN template, with six $\{1\bar{1}00\}$ non-polar facets. While the bottom of the nanohole appears to be pointed or tapered from SEM images, it is represented as being flat for simplicity. Figure 5b shows the real 3D shape of a nanohole created from a $(11\bar{2}2)$ semi-polar GaN template for two different depths. The nanohole geometry is not simply composed of six $\{1\bar{1}00\}$ inclined non-polar facets, as one could have expected, but instead are composed of one $(000\bar{1})$ facet, six $\{1\bar{1}00\}$ non-polar facets having various dimensions and probably a $(11\bar{2}2)$ bottom facet. The colour code of the facets used in Fig. 5b is the same as that used in Fig. 4b,c. As for *c*-plane GaN thermal etching, the $\{1\bar{1}00\}$ non-polar facets are stable. In addition, so is the $(000\bar{1})$ facet. Thermal etching is driven along the $\langle \overline{11}23\rangle$ direction by the expansion of the two lateral $\{1\bar{1}00\}$ non-polar facets and by *c*-plane thermal etching along the $\langle 000\bar{1}\rangle$ direction, and in the opposite direction by the combined expansion of the $(000\bar{1})$ facets (in yellow) and the two bottom $\{1\bar{1}00\}$ non-polar facets (in blue and green) respectively towards the $\langle \bar{1}2\bar{1}0\rangle$ and $\langle 0001\rangle$ direction.

Photoluminescence optical characterization performed on the *c*-plane and semi-polar plane GaN nanohole samples displayed in Figs. 3 and 4a–c, respectively, reveal a change in the NBE intensity and position (see Fig. S5), suggesting improved light absorption/extraction and strain relaxation, which is in agreement with previous reports^14,15,27^.

Different mechanisms have been proposed in the literature to explain GaN decomposition:  decomposition into gaseous Ga and nitrogen, decomposition into liquid Ga and gaseous nitrogen and sublimation of GaN as a diatomic or polymeric product^36,39^.

$$\begin{array}{c}2GaN(s)\to 2Ga(g)+{N}_{2}(g).\\ 2GaN(s)\to 2Ga(l)+{N}_{2}(g)\to 2Ga(g)\,+\,{N}_{2}(g).\\ GaN(s)\to GaN(g)\,or\,{[GaN]}_{x}(g).\end{array}$$

The sublimation of GaN is less likely to be the main mechanism as the presence of H_2_ in almost all the experiments will assist decomposition by the reverse GaN synthesis reaction and the reformation of NH_3_^40,41^.

$$GaN(s)+3/2{H}_{2}(g)\,\to Ga(g)+N{H}_{3}(g).$$

Although no clear formation of Ga droplets was observed in any of the experiments, the decomposition into liquid Ga and gaseous nitrogen cannot be ruled out as the remnants of Ga droplets formed during thermal treatment could be transformed to GaN due to reaction with NH_3_ during the cooling down phase. Ga metal could also accumulate at the GaN/SiN_x_ interface, which could explain the absence of Ga droplets and the robustness of the SiN_x_ mask to buffered oxide etching (Fig. S3). Comparing our experiments to the literature, Koleske *et al*. investigated GaN decomposition as a function of pressure under hydrogen, for a similar range of temperatures as employed in this study^42^. It was reported that for pressures less than 40 Torr (53 mbars) no Ga droplets were observed, which is also consistent with vacuum experiments^39^ and theoretical calculations^40^. The presence of liquid gallium catalyses and enhances GaN decomposition locally on the surface by lowering the activation energy for dissociation^43^, which could explain the significant increase in etch depth observed between 20 mbars (15 Torr) in Fig. 1g and 100 mbars (75 Torr) in Fig. 1e. Therefore, decomposition into liquid Ga and gaseous nitrogen appears to be the driving mechanism for most of our set of SATE experiments, except for two: the one carried out at 20 mbars (Fig. 1g) and the one under pure N_2_ carrier gas (Fig. 1h).

We observe that various annealing parameters influence GaN decomposition and our results for the evolution of the etch rate for a GaN layer covered with SiN_x_ circular nano-openings is in good agreement with previous reports performed on GaN thin films. As such, the GaN thermal etch rate increases as the temperature and pressure increases^36,41,44,45^, as the NH_3_ flow decreases^36,45^, and, with a substitution of carrier gas from pure N_2_ to pure H_2_^36,41,45^.

The mechanism of thermal etching occurring in the direction perpendicular to the c-plane surface has previously been suggested to be defect assisted, preferentially occurring at weak areas such as dislocation sites^17,35^. In our experiments, we observe the additional formation of self-assembled GaN nanopores having various etch depths in between the organized array of GaN nanoholes at high temperatures. The absence of such features for other conditions where strong etching anisotropy is observed suggests that the influence of dislocations can be mitigated by limiting the etching temperature.

Most interestingly, and similarly to the continuous selective area growth (SAG) of nanorods, the anisotropy of *c*-plane GaN thermal etching is largely influenced by the NH_3_ flow rate and the temperature. In continuous SAG, as the NH_3_ flow rate is reduced from hundreds to tens of sccm, the morphology of the nanostructures evolves from nanopyramids, defined by $\{1\bar{1}01\}$ semi-polar planes, to nanorods having six $\{1\bar{1}00\}$ non-polar facets, hence enabling anisotropic growth along the *c*-axis^46–48^. In SATE of nanoholes, a similar behaviour is observed, from the formation of pyramidal pits at high NH_3_ flow rate (Fig. 1f), to the creation of six well-defined $\{1\bar{1}00\}$ non-polar facets for tens of sccm (Fig. 1e). Although the threshold in the NH_3_ flow rate value required to form $\{1\bar{1}00\}$ non-polar facets and enhance anisotropic nanorod growth along the *c*-axis may vary depending on the growth conditions and the configuration of the mask openings^46–48^, it generally remains lower than 50 sccm, with a reduction of the diameter and improved uniformity for values as low as 5–10 sccm^46–48^. In contrast, additional SATE experiments performed at 200 sccm NH_3_ (see Fig. S4) suggest that an NH_3_ flow rate up to hundreds of sccm remains sufficient to obtain highly anisotropic thermal etching. The higher flow rate also leads to a reduction of the nanohole diameter due to the formation of inclined semi-polar planes in the upper part of the nanohole, suggesting that NH_3_ flow rate can also be used to tune the diameter of the nanoholes, as has been observed for nanorods^46–48^. The same analogy can be made with the temperature where the thermal etching anisotropy along the *-c*-axis and growth anisotropy along the *c*-axis are improved with temperature, however, only up to a certain extent since growth and thermal etching will inevitably compete at elevated temperatures for the SAG of nanorods^46–48^.

The formation of facets, e.g. semi-polar or non-polar, is driven by the stability of each surface that depends mainly on the surface energy and the stability of surface atoms. These factors can be understood by simply considering the density of dangling bonds (DBs) per unit area and the surface polarity^49^. Therefore, the relative stability of the facets should follow the order Ga-face> non-polar faces > semi-polar faces > N-face and more precisely $(0001)$ > $\{1\bar{1}00\}$ > $\{11\bar{2}0\}$ > $\{1\bar{1}01\}$ > $\{11\bar{2}2\}$ as the DBs per nm^−2^ are respectively: 11.4 < 12.1 < 14 < 16 < 17.8. However, in most of our SATE experiments, the (0001) surface is efficiently etched away, suggesting, on the contrary, a poor stability of the (0001) surface. There are two potential explanations for this unexpected observation. First, as discussed previously, the presence of liquid gallium can catalyse and enhance GaN decomposition locally on the surface by lowering the activation energy for its dissociation^43^. Second, hydrogen can highly influence the stability of the facets as the large number of unsaturated dangling bonds can be efficiently saturated by the adsorption of hydrogen. Several publications report that a hydrogen environment can efficiently etch GaN via Ga-H and N-H formation at high temperature^50–52^. Additionally, Northrup *et al*. showed that the Ga adatom structure would not be stable if the background pressure of hydrogen is greater than about 10^−12^ atm at T = 1000 K^53^. This corroborates why etching occurs in all experiments except under pure N_2_ carrier gas (Fig. 1h), where the (0001) surface is too stable, with supressed N desorption compared to H_2_ carrier gas^54^. Interestingly, Northrup *et al*. also suggested that, in the presence of hydrogen, a 3(N − H) surface reconstruction forms a passivation layer on the $(000\bar{1})$ surface^53^, which supports the formation and high stability of the $(000\bar{1})$ N-polar surface during the thermal etching of $(11\bar{2}2)$ semi-polar GaN layers (Figs. 4 and 5b). With the (0001) surface stability being reduced either by liquid gallium or Ga-H thermal etching, the next most stable surfaces are the $\{1\bar{1}00\}$ non-polar ones. This analysis agrees well with the identification of six $\{1\bar{1}00\}$ non-polar facets in both the nanoholes created from *c*-plane or $(11\bar{2}2)$ semi-polar GaN layers. Changing the pressure, the temperature or the NH_3_ flow rate will however impact the stability of N-terminated surfaces such as $\{1\bar{1}01\}$ and $\{11\bar{2}2\}$. In our case, semi-polar facets are formed and are stable at low temperature (Fig. 1a), high pressure (Fig. 1c) and high NH_3_ flow rate (Fig. 1f), which follows the trend previously discussed for the SAG of nanorods and for epitaxial lateral overgrowth of GaN^46–49^.

In summary, the key advantages of the SATE are: a highly anisotropic etching, an infinite selectivity of GaN etching over SiN_x_ and SiO_2_ mask etching, the absence of dry etching surface damage and generation of non-radiative recombination centers, and the potential to combine SATE with *in-situ* regrowth without exposure to air (Fig. S6). In addition, SATE of GaN nanoholes via MOVPE appears to be more advantageous than the selective area sublimation of GaN nanoholes by MBE (Fig. S7). The latter displays a relatively low etch rate (≈300 nm/h), hence a longer process time. The nanohole sidewalls are also not faceted with a circularity strongly impacted by defects (Fig. S7e,f). In contrast, the SATE via MOVPE shows a clear faceting of the non-polar sidewalls, with a minimal impact of defects for optimum conditions, and with an etch rate 8 to 20 times higher depending on the conditions employed.

## Conclusions

In this paper, we successfully achieve the selective area thermal etching of highly organized and faceted nanohole structures and discuss the decomposition mechanism and their morphology evolution as a function of the MOVPE environment. A low NH_3_ flow rate and a relatively high temperature combined with a hydrogen environment are key to achieve highly anisotropic thermal etching along the *-c*-axis together with the formation of clear $\{1\bar{1}01\}$ non-polar facets. Selective area thermal etching is not only demonstrated on *c*-plane GaN layers for various filling factors but also on a $(11\bar{2}2)$ semi-polar GaN template. In principle, the process should be transferable to other features than circular mask openings, such as dots, rings or lines, along with other semi-polar orientations.

The simplicity of the thermal etching process on nano-masked GaN templates allows the process to be easily implemented as an annealing step either in a commercial MOVPE growth reactor or alternatively, in a relatively low-cost furnace equipped with hydrogen and ammonia. Combining selective area thermal etching with an additional regrowth step offers the chance to create novel core-shell architectures or to improve the structural and optical quality of III-nitride-based LEDs via filtering extended defects and controlling the directionality of the emission via photonic crystal effects.

## Methods

### MOVPE growth

A single $(11\bar{2}2)$ GaN layer was grown on m-plane sapphire using a high temperature AlN buffer technique by metal organic vapor phase epitaxy (MOVPE). The substrate was initially subjected to thermal cleaning in flowing H_2_, and an atomically flat AlN layer with a thickness of 220 nm was then grown at 1170 °C, followed by the growth of a single layer of GaN with a thickness of 1.3 μm at 1100 °C. More details can be found in our previous publication^55^.

### Dielectric mask fabrication

A SiN_x_ mask was fabricated on a 2-inch *c*-plane GaN and a $(11\bar{2}2)$ semi-polar GaN template. The 30 nm SiN_x_ was first deposited by Plasma Enhanced Chemical Vapor Deposition (PECVD). A ~270 nm bottom antireflective coating (BARC) (Wide 30 W – Brewer Science) layer and a ~360 nm high-contrast positive resist (Dow Ultra-i 123 diluted with Dow EC11 solvent) were spin-coated at 3000 rpm. Circular openings were defined in the resist via Displacement Talbot Lithography (PhableR 100, Eulitha) using a coherent 375 nm light source, an energy density of 1 mW.cm^−2^ and a gap of 150 μm. An integration time between 210 and 270 sec and a Talbot length of 8.81 μm were employed for the 1.5 μm pitch amplitude mask with 800 nm diameter circular openings while an integration time of 60 sec and a Talbot length of 750 nm was employed for the 500 nm pitch phase mask with 300 nm diameter circular openings. After a post-bake at 120 °C for 1 min 30 sec, the resist was developed in MF-CD-26 for 90 sec. The circular openings were then transferred into the BARC and SiN_x_ layers via an inductively coupled plasma dry etch system (Oxford Instruments System 100 Cobra) with the following parameters: a CHF_3_ chemistry of 25 sccm, a temperature set to 20 °C, a pressure of 8 mTorr, 50 RF power and 300 W ICP source power. Finally, the wafer was cleaned in a piranha solution (3:1) to remove the resist and BARC, an oxygen plasma to remove any residue, and a final dip in BOE 100:1 for 10 sec. SEM images of the SiN_x_ openings are shown in Fig. S1.

### Selective area thermal etching (SATE)

SATE was carried out in a 1 × 2′′ horizontal Aixtron MOVPE reactor. The details of the SATE conditions are given in Table 1. Temperature ramp-up has been performed under the same conditions for all experiments, with 3 L N_2_, 4 L of NH_3_ and at 100 mbars in order to avoid any thermal etching of the GaN surface. SATE conditions were then changed once the set temperature was reached. 4 L of NH_3_ was supplied during the ramp-down.

### SEM imaging

Plan-view, cross-section and tilted scanning electron microscope images were acquired using a Hitachi S-4300 SEM.

## Supplementary information

Supplementary Information.
