# Supplementary material for: Influence of the reactor environment on the selective area thermal etching of GaN nanohole arrays
Source: Sci Rep. 2020 Mar 27;10:5642. doi: 10.1038/s41598-020-62539-1 (PMC7101372; doi:10.1038/s41598-020-62539-1)
Supplement: Supplementary file 1 — Supplementary Information. [file 41598_2020_62539_MOESM1_ESM.docx]

Supplementary Information: Influence of the reactor environment on the selective area thermal etching of GaN nanohole arrays

*Pierre-Marie Coulon^*,1^, Peng Feng^2^, Benjamin Damilano^3^, Stéphane Vézian^3^, Tao Wang^2^ and Philip A. Shields^1^*

^1^ Dept. Electrical & Electronic Engineering, University of Bath, Bath, BA2 7AY, UK

^2^ Dept. of Electronic and Electrical Engineering, University of Sheffield, S1 4DE, UK

^3^ Université Côte d’Azur, CNRS, CRHEA, rue B. Gregory, 06560 Valbonne, France

**S1: Dielectric mask fabrication**

Figure S1 shows the hexagonal array of SiN_x_ openings created on *c*-plane GaN template for a pitch of 1.5 µm and 500 nm and on ($11\bar{2}2$) semi-polar GaN layers for a pitch of 1.5 µm. The small distortion and non-uniformity observed for 500 nm pitch SiN_x_ openings (inset in Fig. S1.b) results from the poorer quality of the 500 nm pitch phase mask employed for the Displacement Talbot lithography patterning.


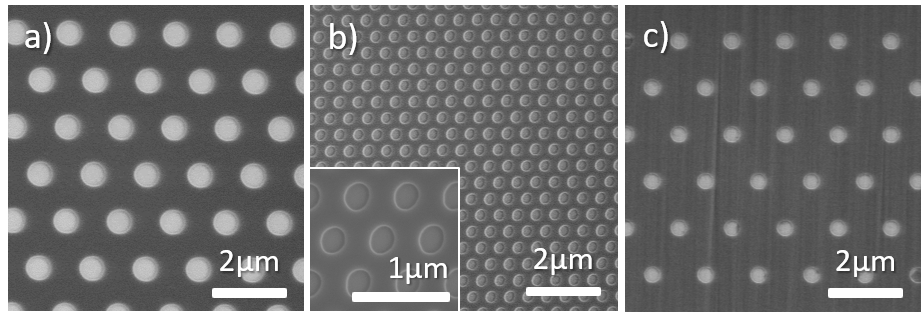


**Figure S1.** Plan view SEM images of SiN_x_ openings on (a-b) *c*-plane GaN template and (c) ($11\bar{2}2$) semi-polar GaN layers.

**S2: Additional selective area thermal etching (SATE) experiments/data**

Figure S2 shows a SATE experiment performed without NH_3_ during 10 min. A shorter time enables to clearly observe the poor anisotropy resulting from thermal etching without NH_3_ along with a significant deterioration of the morphology and dimensions of the nanoholes.

**
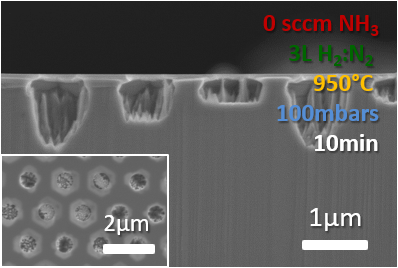
**

**Figure S2.** Cross-section and related plan view (in inset) SEM images of GaN nanoholes after 10 min SATE performed in the same conditions as employed in Figure 1.d.

Figure S3 shows a SEM image of the SiN_x_ mask after SATE experiment and buffered oxide etching (BOE). A clear difference in contrast can be observe at low magnification after 10 min in BOE 5:1. Higher magnification SEM image reveals first, that the 30 nm SiN_x_ mask is not removed after 10 min in BOE 5:1, and second, that the difference in contrast results from a change in morphology of the SiN_x_ surface. Longer BOE time or other wet etch chemistries should be explored to successfully remove the mask while more investigation needs to be performed to understand the nature of the SiN_x_ mask after SATE, which could change into a GaSiN layer.


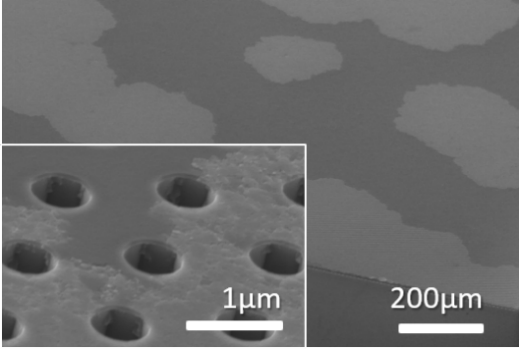


**Figure S3.** 45° tilt SEM images of GaN nanohole after 60 min SATE and 10 minutes in BOE 5:1.

Figure S4 shows a SATE experiment performed with 200 sccm NH_3_. A good thermal etching anisotropy is still achieved for a NH_3_ flow rate of hundreds of sccm. However, semi-polar planes start to form on the upper part of the nanohole, resulting in a decrease of the diameter. This observation suggests that changing the NH_3_ flow rate in the 10 sccm < NH_3_ < 200 sccm range enables to tune the diameter of the nanohole. More investigation needs to be performed to determine the minimum diameter achievable by optimizing the NH_3_ flow rate.

**
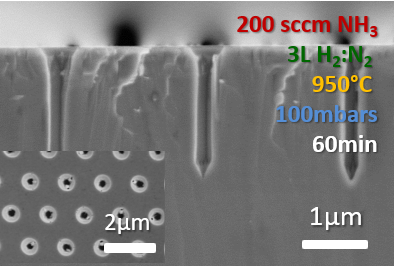
**

**Figure S4.** Cross-section and related plan view (in inset) SEM images of GaN nanoholes after 1h SATE.

**S3: Optical characterisation**

Photoluminescence (PL) experiments were performed at room temperature using a CW frequency-doubled Ar laser at 244 nm with a spot diameter of 120 μm and an excitation power of 30 mW. Figure S5.a and S5.b show the PL for the *c*-plane GaN template and the ($11\bar{2}2$) semi-polar GaN layer, respectively, before and after thermal etching of nanoholes. On *c*-plane GaN, the SATE results in a higher GaN near band edge (NBE) intensity and yellow band intensity. This could suggest a change in the light coupling in and out of the GaN nanohole array. A shift of the NBE position is also observed after SATE which suggest strain relaxation. On ($11\bar{2}2$) semi-polar GaN, the SATE of nanoholes leads to a change in the optical properties between 2.5 and 3.6 eV, with a lower intensity of the band centred around 3 eV, and a clear observation of the NBE. This could suggest a reduction of extended defects after SATE, as they will generally have an impact on non-radiative recombination.


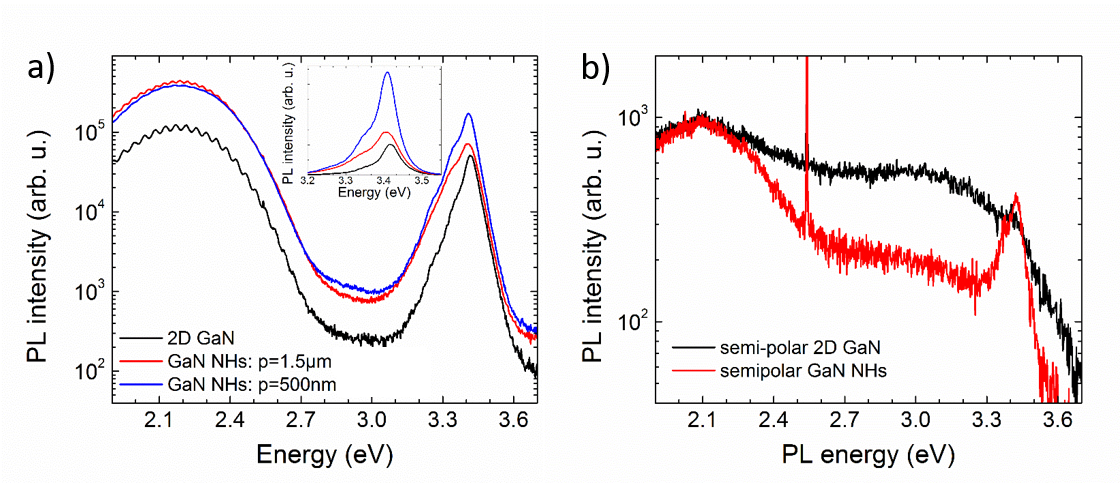


**Figure S5.** PL spectra acquired before and after SATE, for (a) the *c*-plane GaN template and (b) the ($11\bar{2}2$) semi-polar GaN layer.

**S4: Combined SATE and selective area growth (SAG)**

Figure S6 shows a combined SATE and SAG experiment performed on c-plane GaN template for a pitch of 500 nm. SATE was carried out for the same conditions as the one in Figure 2 for 1 h, followed by 30 min SAG under H_2_, a V/III ratio of 7.65 (10 sccm NH_3_ and 15 sccm TMGa), a pressure of 100 mbars and a temperature of 950 °C. Nanorods are successfully regrown on top of each nanoholes. In addition, the regrowth step enables to further smooth out the GaN nanoholes, avoiding the formation of GaN desorption residues in the upper or lower part of the nanohole.


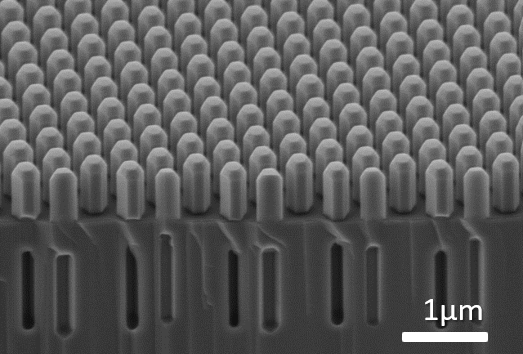


**Figure S6.** Tilted SEM images of combined SATE and SAG experiment.

**S5: Selective area sublimation (SAS) by MBE**

Figure S7 shows SAS experiments performed by MBE on masked GaN surface with SiNx openings having two different diameters. The samples were annealed under vacuum in an MBE chamber during 3 h at 900 °C. The SAS results in a straight nanohole profile with an etch depth of 1 µm. Plan view SEM images reveal a rough nanohole circumference, which is more pronounced for small diameter. This could be due to preferential etching of defects. No clear formation of non-polar facets seems to occur under these conditions.


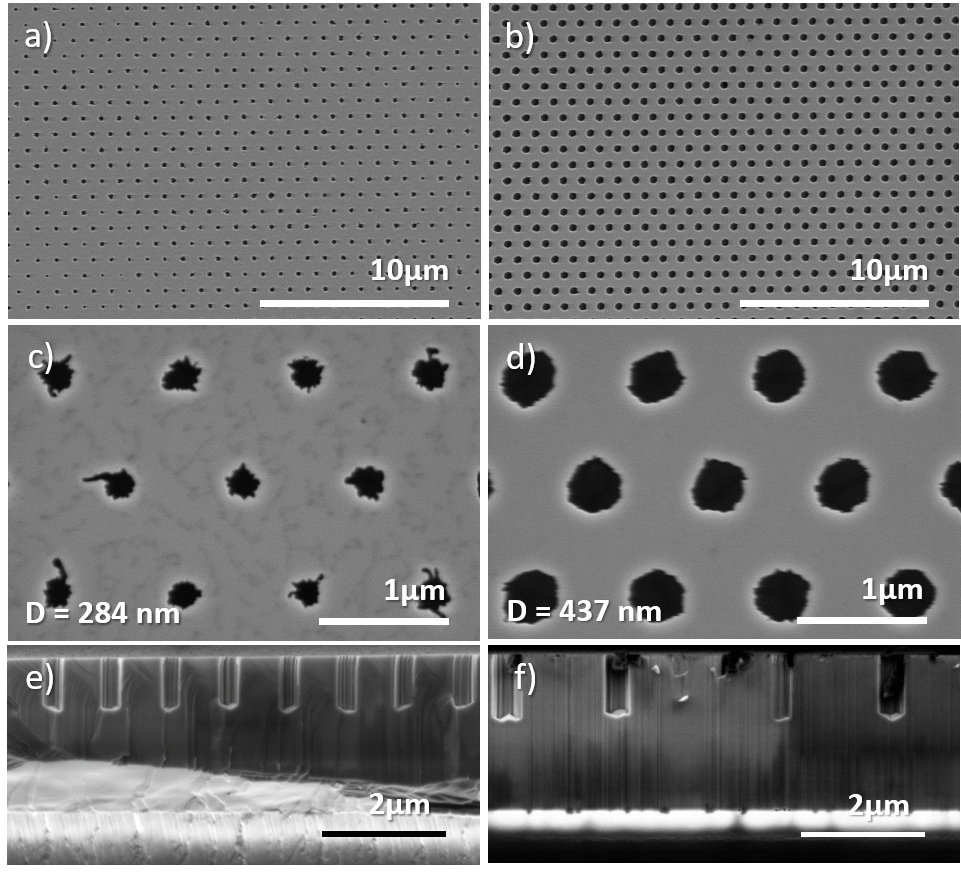


**Figure S7.** Plan view and related cross-section SEM images of GaN nanoholes after 3h SAS.
